# Prognostic value of *EGFR* and *KRAS* in circulating tumor DNA in patients with advanced non-small cell lung cancer: a systematic review and meta-analysis

**DOI:** 10.18632/oncotarget.15412

**Published:** 2017-02-16

**Authors:** Gaowei Fan, Kuo Zhang, Jiansheng Ding, Jinming Li

**Affiliations:** ^1^ National Center for Clinical Laboratories, Beijing Hospital, National Center of Gerontology, Beijing, China; ^2^ Department of Clinical Laboratory, Beijing Chaoyang Hospital, Capital Medical University

**Keywords:** EGFR, KRAS, NSCLC, prognosis, meta-analysis

## Abstract

*EGFR* (exon 19 and exon 21) mutations in patients with advanced non-small cell lung cancer (NSCLC) treated by EGFR-TKIs are associated with a better survival; while *KRAS* mutations predict a worse prognosis. However, there are divergent findings regarding the prognostic value of *EGFR* and *KRAS* mutations in circulating tumor DNA (ctDNA). We aimed to summarize the evidence for the use of circulating *EGFR* and *KRAS* mutations as prognostic factors in advanced NSCLC patients.

We searched the network databases for studies reporting progression-free survival (PFS) and overall survival (OS) stratified by *EGFR* or *KRAS* mutations in ctDNA in advanced NSCLC patients. Thirteen studies enrolling 2,293 patients were reviewed. Correlation of circulating *EGFR* or *KRAS* mutations with patients’ prognosis was assessed by meta-analysis.

The pooled analyses showed that *EGFR* mutations in ctDNA significantly prolong PFS (HR=0.64,95% CI 0.51-0.81, *I*2=0%, *p*=0.0002), namely, in patients treated by EGFR-TKIs. There is a trend to have a prolonged OS for advanced NSCLC patients with circulating *EGFR* mutations who were treated by EGFR-TKIs (HR=0.79, 95% CI 0.52-1.21, *I*2=0, *p*=0.28). *KRAS* mutations detected in ctDNA predict a worse PFS (HR=1.83, 95% CI 1.40-2.40, *p*<0.0001) and OS (HR=2.07, 95% CI 1.54-2.78, *p*<0.00001) in advanced NSCLC patients treated by chemotherapy. Sensitivity analyses and subgroup analyses demonstrated the stability of our conclusion.

Our analysis showed that *EGFR* mutations in ctDNA predicted a better PFS, in particular in advanced NSCLC patients treated by EGFR-TKIs. *KRAS* mutations in ctDNA indicated a worse PFS and OS in patients treated by chemotherapy.

## INTRODUCTION

Non-small cell lung cancer (NSCLC) remains the major cause of cancer-related mortality. Studies showed that epidermal growth factor receptor (EGFR)-tyrosine kinase inhibitors (TKIs) confer better outcome in patients with *EGFR* mutations (exon 19 deletions, exon 21 L858R point mutations) than in those with the wild type [1]. About 5-15% of NSCLC patients harbor *EGFR* mutations [2]. *KRAS* mutations predict worse prognosis among NSCLC patients treated by EGFR-TKIs or chemotherapy [3, 4]. *KRAS* mutations are detected in about 30% of NSCLC in white people [5]. Approximately 97% of *KRAS* mutations in NSCLC involve codon 12 or codon 13 [3, 6]. Several studies performed systematic review and meta-analysis to assess the prognostic value of *EGFR* and *KRAS* mutations in tumor tissue in NSCLC patients [4, 7–9].

Circulating tumor DNA (CtDNA) is shed into the bloodstream by tumor cells [10]. Evidence shows that ctDNA might be used as a noninvasive blood biomarker in tumor medicine [11, 12]. Diagnostic tests for ctDNA such as OncoBEAM^®^ RAS CRC Kit (Sysmex Inostics GmbH), cobas^®^ EGFR Mutation Test V2 (Roche) and EGFR Mutations Detection Kit (AmoyDx) are commercially available for ctDNA detection. The published papers offered divergent findings regarding the prognostic value of *EGFR* and *KRAS* mutations in ctDNA in patients with advanced NSCLC. Only one study by Mao et al. conducted meta-analysis to explore the prognostic value of *EGFR* in ctDNA in advanced NSCLC patients [13]. However, overlapping studies were included in their study [13]. No studies did a systematic review and meta-analysis to assess the prognostic value of *KRAS* mutations in ctDNA in patients with advanced NSCLC. Thus, we conducted a systematic review and meta-analysis to explore their prognostic values in advanced NSCLC patients.

## RESULTS

### Included studies

**Figure 1:**
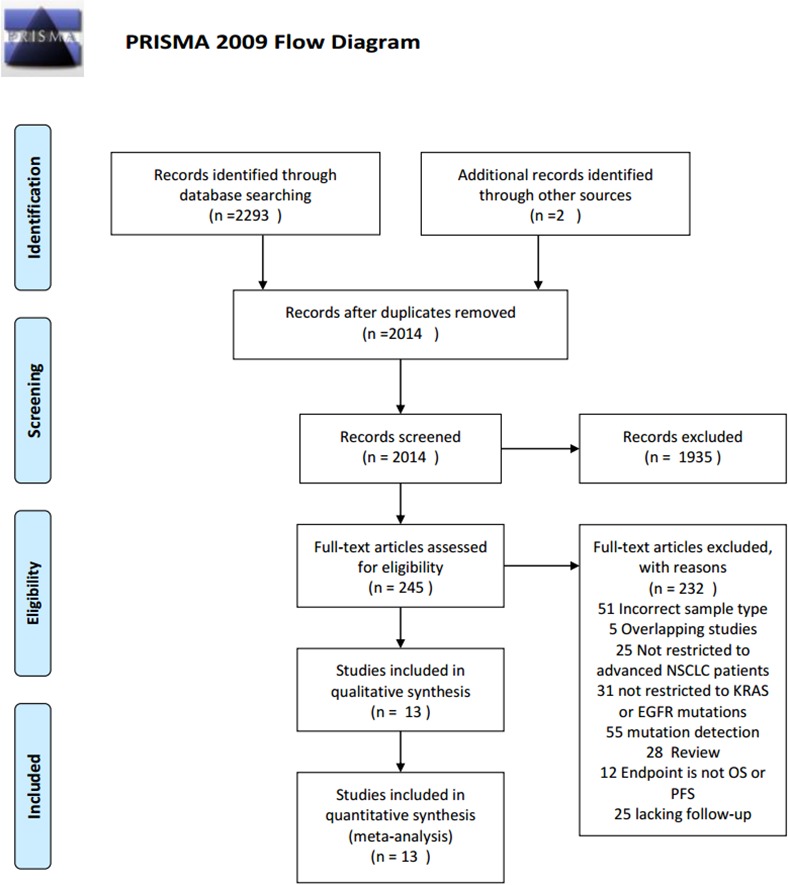
PRISMA 2009 Flow Diagram PRISMA flow diagram for study selection.

A total of 2,295 potential studies were identified. After screening by title and abstract, 2,216 studies were excluded. The main reasons for exclusion were duplicative studies, reviews, not human studies, not relevant to ctDNA, incorrect tumor type and epigenetic alterations. Of the remaining 79 studies, the full text was screened and 66 studies were excluded for lack of follow-up, no information about prognosis, not restricted to advanced NSCLC patients, non-English literature, not restricted to *KRAS* or *EGFR* (exon19 and exon21) mutations. Finally, 13 studies met the inclusion criteria and were included for systematic review and meta-analysis (Figure 1).

### Study characteristics

Thirteen studies containing 1,452 patients were published between 2005 and 2013. These studies analyzed the relationship between circulating *EGFR* and *KRAS* mutations status and survival outcomes. The mean number of patients for each study was 67, ranging from 22 to 308. Four studies were retrospective and 9 were prospective. All studies were published in full and all had enough information to obtain the hazard ratios (HRs) and associated 95% confidence intervals (CIs). The main characteristics of the included publications are summarized in Table 1.

**Table 1:** General characteristics of the study populations

First author name (year)	Country	Publication type	Study design	Patients included in analysis	Male sex N (%)	Median age, y/o	Never smoker/ total	Tumor stage	Treatment	Detection matrix	Sampling time	ctDNA detection	Mutation detection methods	ctDNA positive, n
Mack 2009	USA	Full publication	Pro	45	NR	NR	NR	ad	TKIs	Plasma	Prior to treatment	EGFR/KRAS	DS	6EGFR, 2KRAS
Camps 2005	Spain	Full publication	Pro	67	94	64	7	ad	Chemo	Serum	Prior to treatment	KRAS	DS	20
Camps 2011	Spain	Full publication	retro	308	83.8	60	NR	ad	Chemo	Plasma	Prior to treatment	KRAS	Real-time PCR	27
Huang 2012	China	Full publication	Pro	207	NR	NR	46.4	ad	TKIs	Plasma	Prior to treatment	EGFR	DHPLC	70
Xu 2012	China	Full publication	Pro	51	60.7	54	62.7	ad	gefitinib	Plasma	Prior to treatment	EGFR	ME- Liquidchip	15
Kimura 2007	Japan	Full publication	Retro	42	66.7	58	33.3	ad	gefitinib	Serum	Prior to treatment	EGFR	SARM	7
Kim 2013	South Korea	Full publication	Pro	22	NR	NR	NR	ad	TKIs	Serum	Prior to treatment	EGFR/KRAS	EGFR: PNA-LNA; KRAS: DS	4KRAS ; 5EGFR
Bai 2009	China	Full publication	Pro	102	NR	NR	NR	ad	gefitinib	Plasma	Prior to treatment	EGFR	DHPLC	37
Punnoose 2012	USA/ Australia	Full publication	Retro	37	56.76	NR	19.35	ad	erlotinib/pertuzumab	Plasma	Prior to and during treatment	KRAS/EGFR	SARMS	4EGFR, 5KRAS
Zhuo 2011	China	Full publication	Retro	145	59	M:60.5, W: 62	NR	ad	chemo	Plasma	Prior to treatment	EGFR	DHPLC	54
Qin 2011	China	Full publication	Pro	46	NR	NR	NR	ad	gefitinib	Plasma	NR	EGFR	SARMS	18
Nygaard 2013	Denmark	Full publication	Pro	246	61	66	NR	ad	chemo	Plasma	Prior to treatment	KRAS	In-house real-time PCR	43
He 2009	China	Full publication	Pro	134	6.4	60	NR	ad	surgery/ chemo/ TKIs	Plasma	Prior to treatment	EGFR	DS	66

Abbreviations: Pro: prospective study; Retro: retrospective study; NR: no report; chemo: chemotherapy; ad: advanced stage; DS: direct sequencing; SARMS: Scorpion Amplification Refractory Mutation System; DHPLC: denaturing high-performance liquid chromatography; PNA-LNA: peptide nucleic acid-locked nucleic acid; ME-liquid chip : mutant-enriched liquid chip

## QUALITY ASSESSMENT

**Figure 2:**
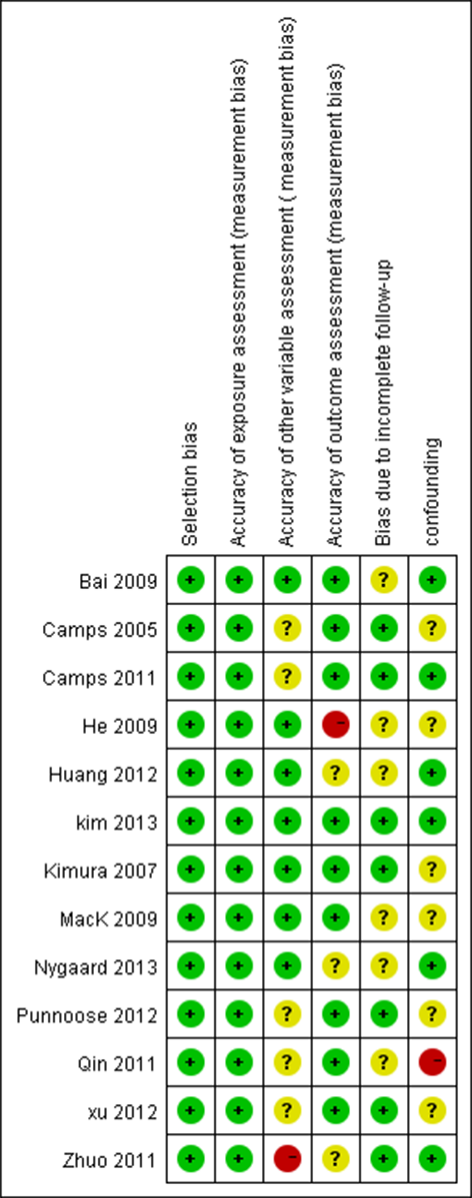
Our judgements about each risk of bias item of included studies Red circles represent studies with high risk of bias; Green circles represent studies with low risk of bias, yellow circles represent studies with uncertain risk of bias.

We assessed risk of bias using the Cochrane Collaboration's tool (the Cochrane Collaboration's tool for assessing risk of bias in randomized trials) [14]. All the included studies had a low risk of bias, as summarized in Figure 2.

### *EGFR* (exon 19 and exon 21) mutations and prognosis

#### Relationship between *EGFR* mutations (exon 19 and exon 21) and PFS

**Figure 3:**
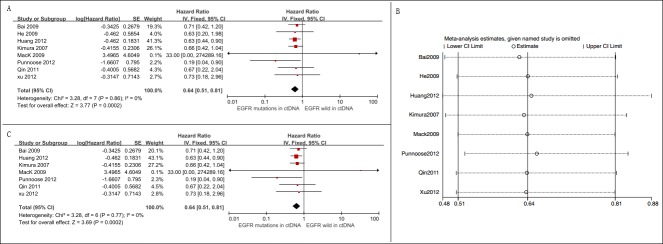
Meta-analysis of the prognosis of circulating *EGFR* mutations for PFS **A.** Forest plots of HR and 95% CI in advanced NSCLC patients. Patients with circulating *EGFR* mutations had a better PFS (HR = 0.64, 95% CI 0.51-0.81); **B**. results of sensitive analysis showed that there was no “dominant” study driving the results of the meta-analysis; **C**. forest plots of HR and 95% CI in advanced NSCLC patients treated by EGFR-TKIs. Circulating *EGFR* mutations indicated a better PFS among patients who were treated by EGFR-TKIs (HR = 0.64, 95% CI 0.51-0.81).

Eight studies assessing the relationship between *EGFR* mutation status in ctDNA and PFS were eligible for the meta-analysis [15–22]. A total of 705 patients were included, and 248 were *EGFR* mutation-positive. Among them, 684 patients (97%) were treated by TKIs, and the rest 21 (3%) were treated by chemotherapy. The overall summary HR was 0.64 (95% CI 0.51-0.81), with a low degree of heterogeneity (*p* = 0.86, *I*2 = 0%). The pooled analysis indicated a better PFS for circulating *EGFR* mutation-positive patients (Figure 3A).

##### Sensitive analysis

Sensitivity analysis by “leave-one-out” strategy showed that there was no “dominant” study driving the results of meta-analysis (Figure 3B).

Six of 8 studies enrolled Asian patients [15, 17, 18, 20–22], one study included both Asian and Western patients [16], and rest one included only Western patients [19]. We changed our criteria by including studies with Asian patients only. The aggregated analysis showed that *EGFR* mutations prolonged PFS among Asian patients (HR = 0.66, 95% CI 0.52-0.83, *I*2 = 0%) (Supplementary Figure S1).

Seven studies examined the relationship between *EGFR* mutations status and PFS among advanced NSCLC patients with TKIs therapy [15–19, 21, 22]. The overall summary HR was 0.64 (95% CI 0.51-0.81, *I*2 = 0%), suggesting that the observed benefit is tightly linked to the effect of EGFR-TKIs in the setting of *EGFR* mutated patients (Figure 3C).

##### Subgroup analysis

We performed subgroup analyses on the basis of detection matrix (serum *vs*. plasma) and study year (prior to 2010 *vs*. after 2010). There was no statistical significance between these subgroups (Table 2).

**Table 2:** Subgroup analyses on the basis of detection matrix and study year for *EGFR* mutations and progression-free survival analysis

Trial characteristic	Subgroup analysis					
	Stratification variable	Number of study arms	Pooled hazard ratios	95% CI	*p*-value within subgroups	*p*-value between subgroups
Detection matrix	Serum	1	0.66	0.42–1.04	0.07	0.89
	Plasma	7	0.64	0.51–0.81	0.78	
Study year	Before 2010	4	0.68	0.49–0.94	0.86	0.62
	After 2010	4	0.61	0.44–0.84	0.52	

#### Relationship between *EGFR* (exon 19 and exon 21) mutations and overall survival

**Figure 4:**
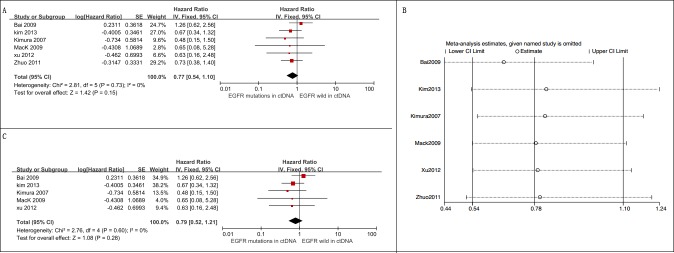
Meta-analysis of the prognosis of circulating *EGFR* mutations for OS **A**. Forest plots of HR and 95% CI in advanced NSCLC patients. Only marginally statistically significant OS (HR = 0.77, 95% CI 0.54-1.10) was observed between NSCLC patients with and without circulating *EGFR* mutations; **B**. results of sensitive analysis showed that there was no “dominant” study driving the results of the meta-analysis; **C**. forest plots of HR and 95% CI in advanced NSCLC patients treated by EGFR-TKIs, there was no statistical significance between patients with and without circulating *EGFR* mutations (HR = 0.79, 95% CI 0.52-1.21, *p* = 0.15).

Six studies with 407 patients examining the relationship between circulating *EGFR* mutation status and OS among advanced NSCLC patients were included [15, 19, 21–24]. Among them, 124 were circulating *EGFR* mutation-positive. Our pooled analysis showed that there is a trend for longer OS in patients harboring circulating *EGFR* mutations (HR = 0.77, 95% CI 0.54-1.10, *I*2 = 0%) (Figure 4A).

##### Sensitive analysis

The “leave-one-out” strategy demonstrated that there was no dominant study driving the results of meta-analysis (Figure 4B).

Five of 6 studies enrolled patients from Asia [15, 21–23, 25]; only 1 study included patients from the West [19]. We changed our criteria by including studies with Asian patients. This change did not alter our conclusion (HR = 0.78, 95% CI 0.54-1.11, *I*2 = 0%) (Supplementary Figure S2).

Five studies reported the relationship between *EGFR* mutation status and OS among advanced NSCLC patients treated by TKIs [15, 19, 21, 22, 25]. The pooled analysis revealed that in the setting of EGFR-TKIs treatment, *EGFR*-mutation positive patients trend to have a longer OS (HR = 0.79, 95% CI 0.52-1.21, *I*2 = 0) (Figure 4C).

##### Subgroup analysis

Subgroup analyses on the basis of detection matrix (serum *vs*. plasma) and study year (prior to 2010 *vs*. after 2010) were performed. There was no statistical significance between these subgroups (Table 3).

**Table 3:** Subgroup analyses on the basis of detection matrix and study year for *EGFR* mutation and overall survival analysis

Trial characteristic	Subgroup analysis					
	Stratification variable	Number of study arms	Pooled hazard ratios	95% CI	*p*-value within subgroups	*p*-value between subgroups
Detection matrix	Serum	2	0.61	0.34–1.10	0.62	0.33
	Plasma	4	5.13	2.43–10.82	0.66	
Study year	Before 2010	3	0.93	0.52–1.67	0.35	0.42
	After 2010	3	0.69	0.44–1.08	0.97	

### *KRAS* mutations and prognosis

#### Relationship between *KRAS* mutations in ctDNA and PFS

**Figure 5:**
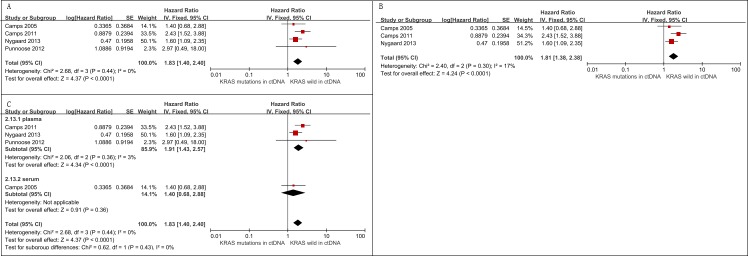
Meta-analysis of the prognosis of circulating *KRAS* mutations for PFS **A**. Forest plots of HR and 95% CI in advanced NSCLC patients. The pooled analysis showed that circulating *KRAS* mutations were associated with a worse PFS (HR = 1.83, 95% CI 1.40-2.40); **B**. forest plots of HR and 95% CI in advanced NSCLC patients treated with chemotherapy. Circulating *KRAS* mutations were associated with a shorter PFS among patients treated by chemotherapy (HR = 1.81, 95% CI 1.38-2.38); **C**. Subgroup analyses on the basis of detection matrix (serum *vs* plasma) indicates no statistical significance between these *KRAS* mutations detected in serum and *KRAS* mutations detected in plasma regarding PFS (*p* = 0.43).

Four studies with 658 patients assessing the relationship between *KRAS* mutations in ctDNA and PFS among advanced NSCLC patients were eligible for meta-analysis [3, 6, 16, 24]. Among them, 95 were circulating *KRAS* mutation-positive. In this analysis, *KRAS* mutations in ctDNA were associated with a worse PFS (HR = 1.83, 95% CI 1.40-2.40, *I*2 = 0) (Figure 5A).

##### Sensitive analysis

Three out of 4 studies explored *KRAS* mutations in ctDNA as a predictive factor for the effect of chemotherapy on advanced NSCLC patients [3, 6, 24]. The pooled analysis showed that *KRAS* mutations in ctDNA indicate a worse PFS in patients treated by chemotherapy (HR = 1.81, 95% CI 1.38-2.38, *I*2 = 17%) (Figure 5B).

The “leave-one-out” strategy showed that there was no dominant study driving the results of meta-analysis (Supplementary Figure S3)

##### Subgroup analysis

We performed subgroup analysis based on detection sample (serum *vs*. plasma). There was no statistical significance between these subgroups (Figure 5C).

#### Relationship between *KRAS* mutations in ctDNA and OS

**Figure 6:**
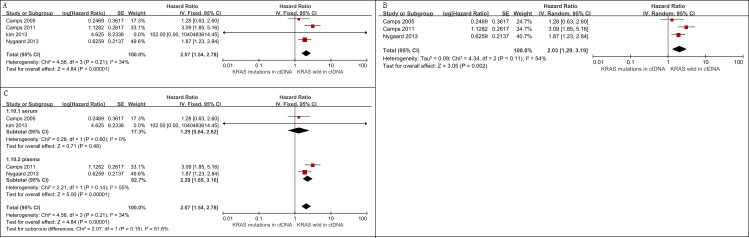
Meta-analysis of the prognosis of circulating *KRAS* mutations for OS **A**. Forest plots of HR and 95% CI in advanced NSCLC patients; patients with circulating *KRAS* mutations had a worse OS (HR = 2.07, 95% CI 1.54-2.78) **B**. forest plots of HR and 95% CI in advanced NSCLC patients treated with chemotherapy; circulating *KRAS* mutations were associated with a worse OS among patients treated by chemotherapy (HR = 2.03, 95% CI 1.29-3.19) **C**. subgroup analysis in advanced NSCLC patients on the basis of detecting matrix (serum *vs*. plasma). The subgroup difference was not significant (*p* = 0.43).

Five studies assessed the relationship between *KRAS* mutation status and OS among advanced NSCLC patients [3, 6, 24–26]. A total of 693 patients were included and 106 were *KRAS* mutation-positive. Findings from the meta-analysis suggested that *KRAS* mutations in ctDNA were associated with an unfavorable OS (HR = 2.07, 95% CI 1.54-2.78, *I*2 = 34%) (Figure 6A).

##### Sensitive analysis

Three of 4 studies examined *KRAS* mutations in ctDNA as a predictive factor among advanced NSCLC patients who were treated with chemotherapy [3, 6, 24]. Our pooled analysis showed that patients with *KRAS* mutations in ctDNA had a worse OS (HR = 2.03, 95% CI 1.29-3.19, *I*2 = 54%) (Figure 6B).

The “leave-one-out” sensitivity analysis showed that our results were stable (Supplementary Figure S4).

##### Subgroup analysis

We performed subgroup analysis based on detection sample (serum *vs*. plasma). The pooled analysis demonstrated that the association between OS and *KRAS* mutation status was slightly stronger in plasma (HR = 2.29, 95% CI 1.65-3.16) than in serum (HR = 1.29, 95% CI 0.64-2.62), though the subgroup difference was not significant (*p* = 0.15) (Figure 6C).

## DISCUSSION

*EGFR* genotyping has become a routine test for the selection of patients as candidates for TKIs therapy. *KRAS* mutations test may help to predict less benefit of treatment with EGFR-TKIs and chemotherapy.

*EGFR* and *KRAS* mutation tests are often carried out from formalin-fixed paraffin embedded (FFPE) tumor tissue samples. However, molecular testing with FFPE has some limits. DNA extracted from FFPE is fragmented and also contains DNA lesions (including uracil and thymine deriving from cytosine deamination) that may lead to sequence artifacts [27]. Frozen tumor biopsies and fresh biopsies may complement FFPE. However, sometimes this is not feasible in clinical practice for advanced NSCLC, especially for successive dynamic monitoring. Tumor heterogeneity is another hurdle when utilizing tissue samples for mutation tests [28]. CtDNA may overcome these limits, and may be used as a surrogate [29]. However, we still have a limited understanding of the origination of ctDNA. If the ctDNA analyzed originates from apoptotic/necrotic tumor cells, it may have limited applicability for prognostic analysis. If the ctDNA utilized is from actively shedding tumor cells (either in circulation or in primary/metastatic sites), it may provide valuable information for treatment decision and prognostic prediction. Due to tumor heterogeneity, the choice of target treatment should ideally be based on the ctDNA from metastatic sites instead of on that from the primary sites [30]. Therefore, the limited understanding of its origination arises the question whether *EGFR* or *KRAS* mutations detected in ctDNA could be used as biomarkers for prognosis prediction.

The current meta-analysis established that advanced NSCLC patients with ctDNA *EGFR* mutations predicted a better PFS, namely, in patients treated by EGFR-TKIs. There is a trend to have a prolonged OS for advanced NSCLC patients with ctDNA *EGFR* mutations who were treated by EGFR-TKIs. Mao et al. carried out meta-analysis in advanced NSCLC patients treated by TKIs and arrived at a different conclusion. Their analyses showed that *EGFR* mutations detected in blood were associated with better OS (HR = 0.71, 95% CI 0.50-0.99, *p* = 0.61) [13]. The difference was likely to be caused by including suspected overlapping studies in that study [13]. Moreover, most of the HR estimates were extrapolated from the survival curves, which also contributed to this discrepancy. Huang et al. and Lee et al. carried out meta-analyses in NSCLC patients with tissue *EGFR* mutations. They found that the improvement in OS was only marginally statistically significant in patients receiving TKIs therapy, which was similar to our conclusion [7, 8].

Our meta-analysis showed that at least in patients treated by chemotherapy, circulating *KRAS* mutations correlated with worse PFS and OS. This finding was consistent with the meta-analysis conducted by Chen et al, who evaluated the prognostic value of tissue *KRAS* mutation status [4].

Either serum or plasma was used as detecting matrix for genotyping in the eligible studies. The amounts of cell-free DNA are much higher in serum due to cell lysis during sample processing, hence reducing the fraction of tumor DNA in serum. However, this discrepancy did not alter our conclusions.

This meta-analysis had some advantages. First, we performed a comprehensive review and reported the most up-to-date published data. Second, no heterogeneity was found in this meta-analysis. Finally, this was the first meta-analysis to assess the prognostic value of circulating *KRAS* mutations in advanced NSCLC patients.

Despite our efforts to provide an accurate and comprehensive analysis, the limitations of our meta-analysis should be highlighted. First, *EGFR* exon 19 and exon 21 respond differently to TKIs treatment [9]; thus, it is necessary to perform subgroup analysis according to *EGFR* mutation subtypes in a future study. Second, we did not perform subgroup analyses based on age, sex, smoking status, and detection methods due to insufficient data. Another limitation of our meta-analysis is that the status of other actionable mutations such as *ALK* rearrangements were not considered and should be included in an analysis with more available data in future studies. Finally, the eligible studies only performed univariate analyses; we cannot infer from our meta-analysis whether *EGFR* or *KRAS* mutations in ctDNA could be an independent factor or not.

Selection bias may exist in our paper. For *EGFR* and *KRAS* involved in lung cancers, striking differences in molecular alterations of these genes have been found in never and ever smokers [31]. Epidemiological studies of lung cancer showed that *EGFR* mutations occur more frequently in never smoker East Asia, while *KRAS* mutations occur more frequently in smokers and less common in never smoker East Asia [30]. In our paper, the rate of *EGFR* mutations is higher than usually expected, and the rate of *KRAS* mutations is much lower in the selected studies. This may be due to a high rate of never-smoker patients in our study.

Despite the aforementioned limitations, this meta-analysis suggested that *EGFR* mutations detected in ctDNA were associated with a better PFS, namely, in patients treated by EGFR-TIKs. There is a trend to have a prolonged OS for patients with ctDNA *EGFR* mutations who were treated by EGFR-TKIs. Circulating *KRAS* mutation-positive status in advanced NSCLC predicts a worse PFS and OS in patients treated by chemotherapy.

## MATERIALS AND METHODS

### Search methods for identification of studies

Meta-analysis Of Observational Studies in Epidemiology (MOOSE) guidelines were conformed to identify potential relevant studies. We did systematic electronic searches of Medline, Embase, Web of Science, the Cochrane Library, and Scopus up to October 10, 2015 (no start date limit was applied). The search strategy used was as follows: “Carcinoma, Non Small Cell Lung”, “Carcinomas, Non-Small-Cell Lung”, “Lung Carcinoma, Non-Small-Cell”, “Lung Carcinomas, Non-Small-Cell”, “Non-Small-Cell Lung Carcinomas”, “Non small Cell Lung Cancer”, “Non-Small-Cell Lung Carcinoma”, “Non Small Cell Lung Carcinoma”, “Carcinoma, Non-Small Cell Lung”, “NSCLC”, “Non-Small Cell Lung Cancer”, “ctDNA”, “circulating tumor DNA”, “cell free DNA”, “serum DNA”, “plasma DNA”, “circulating DNA, free DNA”, “free DNA”, “cfDNA”, “prognosis”, “survival”, “prognostic”, “predictive”. Relevant MeSH (Medline) or Emtree (Embase) terms were used where possible. We also hand searched the relevant reference lists to identify new studies. Conference posters and letters that fulfilled the inclusion criteria were also included to capture grey literature. The literature search was confined to English publications.

Two investigators (Fan GW and Zhang K) independently assessed each study for inclusion, and discrepancies were resolved by discussion. Whenever overlapping samples existed (e.g., same authors, overlapping period of study, same protocol ID, overlapping patients), we retained the report with the largest patient population.

### Criteria for considering studies for this review

Eligible studies met the following criteria:

1. dealt with advanced NSCLC (stage IIIB or IV) patients only;
2. analyzed the correlation between patient survival and *EGFR* mutations (exon 19 deletions or L858R) and/or *KRAS* mutations in ctDNA;
3. had follow-up for overall survival (OS) and/or progression-free survival (PFS); and
4. provided enough information to obtain HRs directly or indirectly.

Both prospective and retrospective studies were included. Reviews, comments, and case reports were excluded. Studies with less than five patients were also excluded.

### Data extraction

Two investigators (Fan GW and Ding JS) independently screened the eligible studies and extracted data using a predefined information sheet that included the following information: first author, publication year, country where the study was conducted, publication type, study design, patients included in analysis, median age, percentage of males, percentage of non-smokers, tumor stage, treatment, detection sample, the time of sampling, ctDNA detection, detection methods, and number of patients with positive ctDNA. Discrepancies were resolved by discussion.

### Quality assessment

We evaluated the quality of included studies using the Cochrane Collaboration's tool for assessing risk of bias [32]. Specifically, studies were judged on (1) selection bias: studies that had an explicit statement of inclusion and exclusion criteria were rated as low risk of selection bias; (2) accuracy of exposure assessment, also called measure bias: studies with an explicit statement regarding ctDNA detection methods were rated as low risk; (3) accuracy of other variable assessment: other molecular alterations such as epigenetic alterations and *EGFR* T790M also influence the prognosis of NSCLC patients; (4) accuracy of outcome assessment; (5) bias due to incomplete follow-up (e.g., median follow-up length, range, and loss-to-follow-up rate were satisfactorily reported); (6) confounding, which included known or commonly discussed confounders in the relationship between ctDNA and survival, such as age, smoking, or other factors that were adjusted.

### Measures of treatment effect

The primary outcome was PFS. The secondary outcome was OS. Two investigators (Fan GW, Zhang K) extracted the HRs and their 95% confidence interval (95% CI) to assess the prognostic value of *EGFR* and *KRAS* mutations in ctDNA. If HRs for ctDNA were not available, we calculated them indirectly using the methods of Parmar [33]. By convention, HR = 1 indicates a lack of association between ctDNA status and prognosis; HR > 1 indicates a worse survival for patients with ctDNA positive; HR < 1 represents a benefit outcome for the ctDNA-positive group.

### Statistical analysis

We used the chi-square test to detect heterogeneity and *I*^2^ statistic to measure heterogeneity. A *p* value > 0.10 and an *I*^2^ < 50% indicated a lack of significant heterogeneity; then, the fixed-effects model was used to calculate the pooled HR. Otherwise, the random-effects model was adopted. The effect of ctDNA status on survival was considered statistically significant if the 95% CI for the overall HR estimate did not contain 1.

Sensitivity analyses were conducted for the meta-analysis to check for stability of the overall results. Subgroup analyses were performed using a random-effects model because of the diverse clinical heterogeneity.

Analysis was carried out using Review Manager 5.3 and Stata 12.

### Ethics statement

This study was a literature-based study and no ethics approval was needed.

## SUPPLEMENTARY MATERIALS FIGURES


