# Supplementary material for: Prognostic value of EGFR and KRAS in circulating tumor DNA in patients with advanced non-small cell lung cancer: a systematic review and meta-analysis
Source: Oncotarget. 2017 Feb 16;8(20):33922–32. doi: 10.18632/oncotarget.15412 (PMC5464923; doi:10.18632/oncotarget.15412)
Supplement: Supplementary file 1 [file oncotarget-08-33922-s001.pdf]

Prognostic value of *EGFR* and *KRAS* in circulating tumor DNA in patients with advanced non-small cell lung cancer: a systematic review and meta-analysis

Supplementary Material

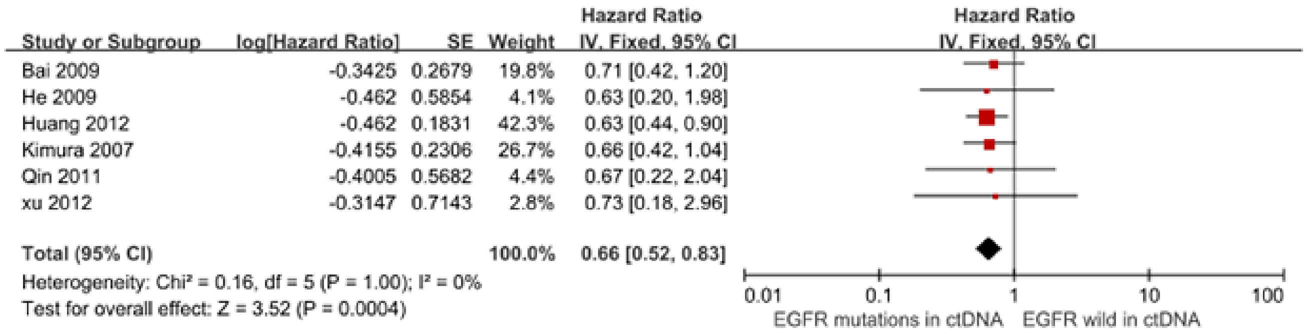

Supplementary Figure S1: Association between PFS and circulating EGFR mutations in Asian patients. Circulating EGFR mutations were associated with a better PFS among Asian patients (HR=0.66, 95% CI 0.52-0.83).

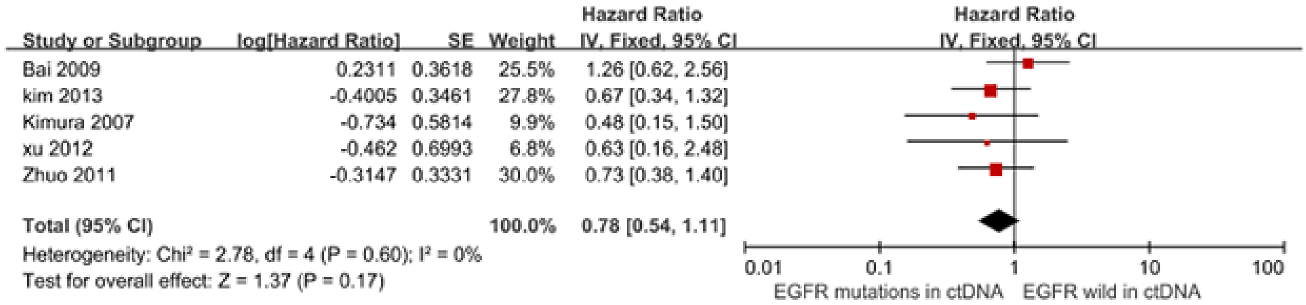

Supplementary Figure S2: Association between OS and circulating EGFR mutations in Asian patients. Circulating EGFR mutations were associated with a better OS among Asian patients, however, there was no statistical significance (HR=0.78, 95% CI 0.54-1.11).

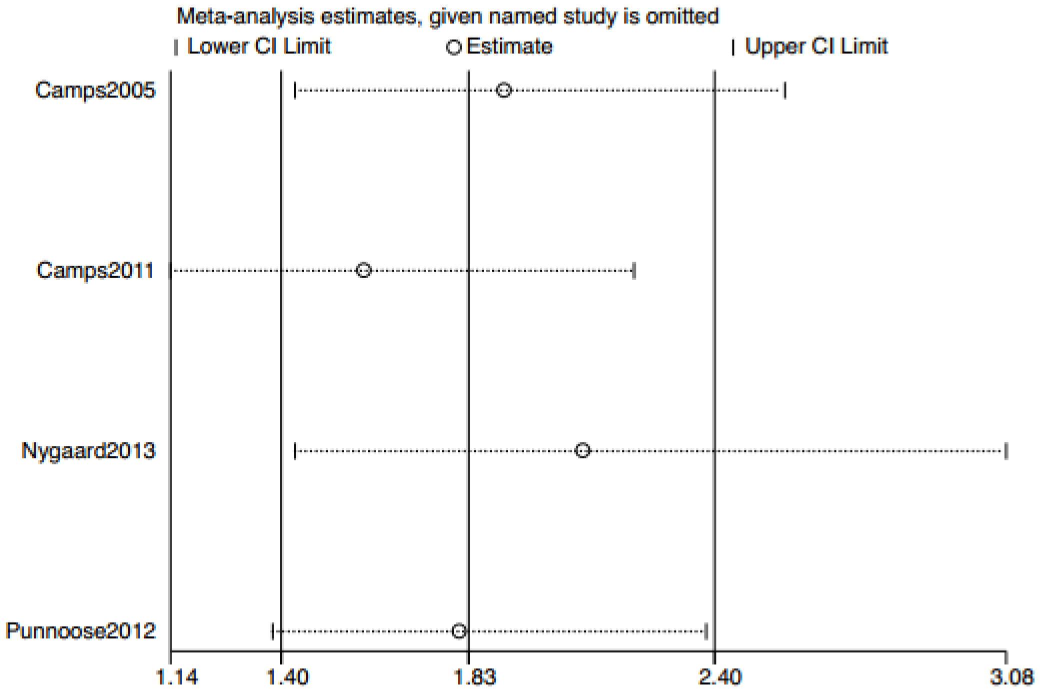

Supplementary Figure S3: Meta-analysis of the prognosis of circulating KRAS mutations for PFS. Sensitivity analysis by "leave-one-out" strategy shows that there was no dominant study driving the results of meta-analysis.

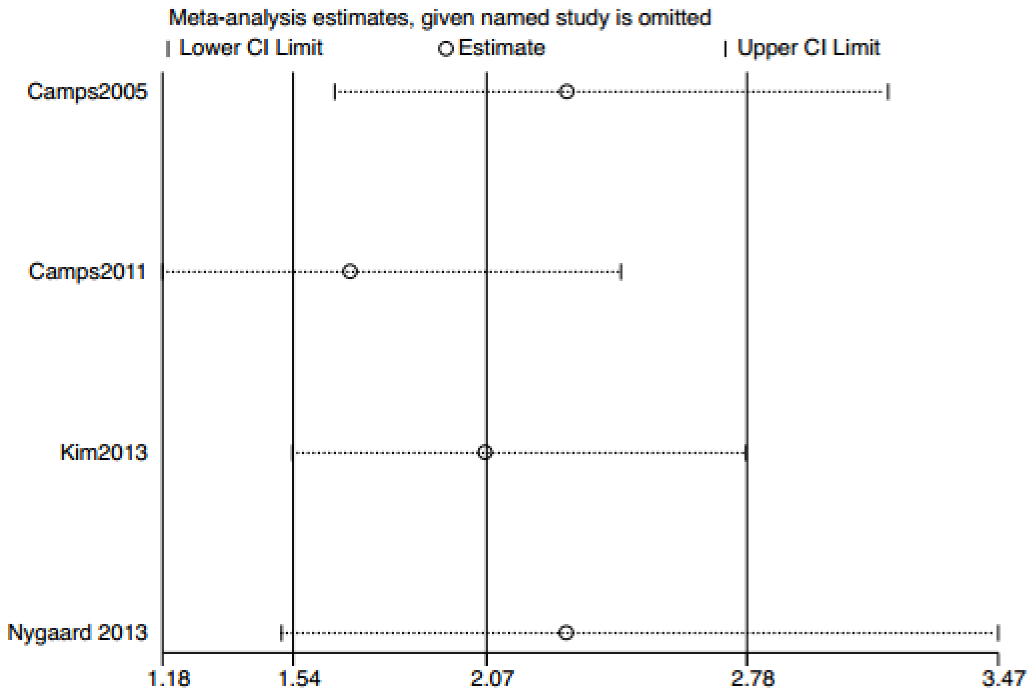

Supplementary Figure S4: Meta-analysis of the prognosis of circulating KRAS mutations for OS. Sensitivity analysis by "leave-one-out" strategy shows that there was no dominant study driving the results of meta-analysis.
